# Drug-coated balloons in treatment of in-stent restenosis: a meta-analysis of randomised controlled trials

**DOI:** 10.1007/s00392-012-0532-3

**Published:** 2012-12-20

**Authors:** Eliano Pio Navarese, David Austin, Paul A. Gurbel, Felicita Andreotti, Udaya Tantry, Stefan James, Antonino Buffon, Marek Kozinski, Karolina Obonska, Kevin Bliden, Young-Hoon Jeong, Jacek Kubica, Vijay Kunadian

**Affiliations:** 1 Department of Cardiology and Internal Medicine, Ludwik Rydygier Collegium Medicum, Nicolaus Copernicus University, Skłodowskiej-Curie Street No 9, 85-094 Bydgoszcz, Poland; 2 Freeman Hospital, Newcastle Upon Tyne Hospitals NHS Foundation Trust, Newcastle upon Tyne, UK; 3 Sinai Center for Thrombosis Research, Sinai Hospital of Baltimore, Baltimore, MD US; 4 Department of Cardiovascular Medicine, Catholic University of the Sacred Heart, Rome, Italy; 5 Uppsala Clinical Research Center, Uppsala University, Uppsala, Sweden; 6 Division of Cardiology, Department of Internal Medicine, Gyeongsang National University Hospital, Jinju, Korea; 7 Institute of Cellular Medicine, Faculty of Medical Sciences, Newcastle University, Newcastle upon Tyne, UK

**Keywords:** Coronary artery disease, Angioplasty, Drug-coated balloons, Drug-eluting stents, Meta-analysis, Randomized controlled trials

## Abstract

**Background:**

Drug-coated balloons (DCBs) have been developed for the percutaneous treatment of coronary artery disease. An initial focus has been the management of in-stent restenosis (ISR) but randomised controlled trials (RCTs) have been small and powered only for angiographic endpoints.

**Objective:**

The aim of the work was to assess the clinical and angiographic outcomes of patients treated for ISR with DCB versus control (balloon angioplasty or drug-eluting stents) by a meta-analysis of RCTs.

**Methods:**

A comprehensive search was performed of RCTs where patients with ISR were randomly assigned to either DCB or alternative coronary intervention. Outcome measurements were death, myocardial infarction (MI), target lesion revascularisation (TLR), binary definition of restenosis and in-lesion late luminal loss (LLL).

**Results:**

Four studies were identified that fulfilled the inclusion criteria. Pooled odds ratios (ORs) were calculated for patients treated for ISR (*n* = 399). Mean follow-up duration was 14.5 months. DCBs were associated with lower rates of TLR [8.8 vs. 29.7 % OR (95 % confidence interval, CI) 0.20 (0.11–0.36), *p* < 0.0001], binary restenosis [10.3 vs. 41.3 % OR (95 % CI) 0.13 (0.07–0.24), *p* < 0.00001] and MI [0.5 vs. 3.8 %, OR (95 % CI) 0.21 (0.04–1.00), *p* = 0.05]. No significant heterogeneity was identified.

**Conclusion:**

Drug-coated balloons appear to be effective versus control in reducing TLR and possibly MI versus balloon angioplasty or drug-eluting stents in the management of ISR.

## Introduction

Several drug-coated balloons (DCBs) have been developed for the percutaneous treatment of coronary artery disease. They are all coated with the anti-proliferative drug paclitaxel with or without a carrier that influence not only drug solubility but also drug transfer and biological efficacy. In practise, the DCB is inflated within the coronary artery, with direct drug delivery to the coronary endothelium [1]. The aim of local drug delivery is to inhibit neo-intimal hyperplasia and promote rapid healing of the treated vessel. In comparison with drug eluting stents (DES), the current gold standard strategy for percutaneous coronary intervention (PCI), DCBs have theoretical benefits that include more uniform drug distribution at higher doses, no permanent vascular scaffold left in situ and no need for a polymer. These features allow for shorter durations of dual anti-platelet therapy and may eliminate some of the stimuli that predispose to stent thrombosis [1, 2].

With the widespread successful application of DES [3], it is not currently clear where DCBs may provide additional benefits. Restenosis within previously implanted bare-metal stents (BMS) or DES is considered a possible indication for DCB therapy [4]. Several randomised controlled trials (RCTs) have been performed, but they have been small and powered for angiographic endpoints.

The goal of this meta-analysis was to determine the role of DCB in the management of in-stent restenosis (ISR).

## Methods

The present meta-analysis was performed according to the established methods of Cochrane Guidelines [5] and in compliance with the PRISMA statement [6] for reporting systematic reviews and meta-analyses in health care interventions.

### Study eligibility and search strategy

Studies were eligible for inclusion if patients were randomly assigned to either DCB or an alternative coronary intervention, with appropriate reporting of methodologies, baseline patient and procedural data and clinical events at least 6 months following the index procedure. Published manuscripts and adequately reported oral abstracts were considered for inclusion to minimise the risk of publication bias. No language barrier was applied. Medline, Embase, and Cochrane databases were searched, as well as the web-based resources, ‘clinicaltrials.gov’ and Google Scholar. Search terms were “drug eluting balloon”, “drug coated balloon”, “paclitaxel eluting balloon”, “randomised controlled trial”, “controlled clinical trial”, “double-blind”, “placebo”, and “random”. Where unpublished RCTs were identified, Scientific Sessions of the American College of Cardiology [http://www.acc.org], American Heart Association [http://www.aha.org], European Society of Cardiology [http://www.escardio.org], Transcatheter Cardiovascular Therapeutics [http://www.tctmd.com] and EuroPCR [http://www.europcr.com] websites were searched. The literature searches were performed independently by two authors. Titles and abstracts were reviewed to determine appropriateness for further review. Full texts of studies of potential interest were then retrieved. Only studies of restenosis were included in this meta-analysis.

### Clinical outcomes and definitions

Data were extracted on baseline variables and on clinical and angiographic outcomes. Outcomes of interest defined a priori were all-cause mortality, myocardial infarction (MI, defined according to each study protocol), target lesion revascularisation (TLR, defined as re-intervention on the index treated lesion), binary restenosis (defined as ≥50 % luminal diameter stenosis by quantitative coronary angiography), and in-lesion late luminal loss (LLL). LLL was the difference between the in-segment minimal lumen diameter after the procedure and at angiographic follow-up, as evaluated by quantitative coronary angiography.

### Statistical analysis

Odds ratio (OR) and 95 % confidence interval (95 % CI) were used as summary statistics for binary data, whereas mean difference (MD) was used for continuous data. Heterogeneity was assessed by Cochran’s *Q* test, with a 2-tailed *p* = 0.1. The statistical inconsistency test (*I*2) {[(*Q*−*df*)/*Q*] × 100 %, where *Q* is the *chi*-squared statistic and *df* its degrees of freedom} was also employed to overcome the low statistical power of Cochran’s *Q* test. Pooled ORs were calculated using a Fixed Effect Model with the Mantel–Haenszel method. The DerSimonian and Laird Random Effects Model was used in case of significant heterogeneity and/or moderate or significant inconsistency (>50 %) across studies. The potential publication bias was examined by constructing a ‘funnel plot’, in which the standard error (SE) of the ln OR was plotted against the OR of the chosen outcome. Finally, we addressed the influence of each study by testing whether, deleting each in turn, would have changed significantly the pooled results of the meta-analysis (sensitivity analysis). Review Manager 5.1 (The Nordic Cochrane Center, Købehvn, Denmark) and SPSS for Windows version 15 (SPSS, Chicago, Illinois) were used for statistical computations.

## Results

### Literature search

**Fig. 1:**
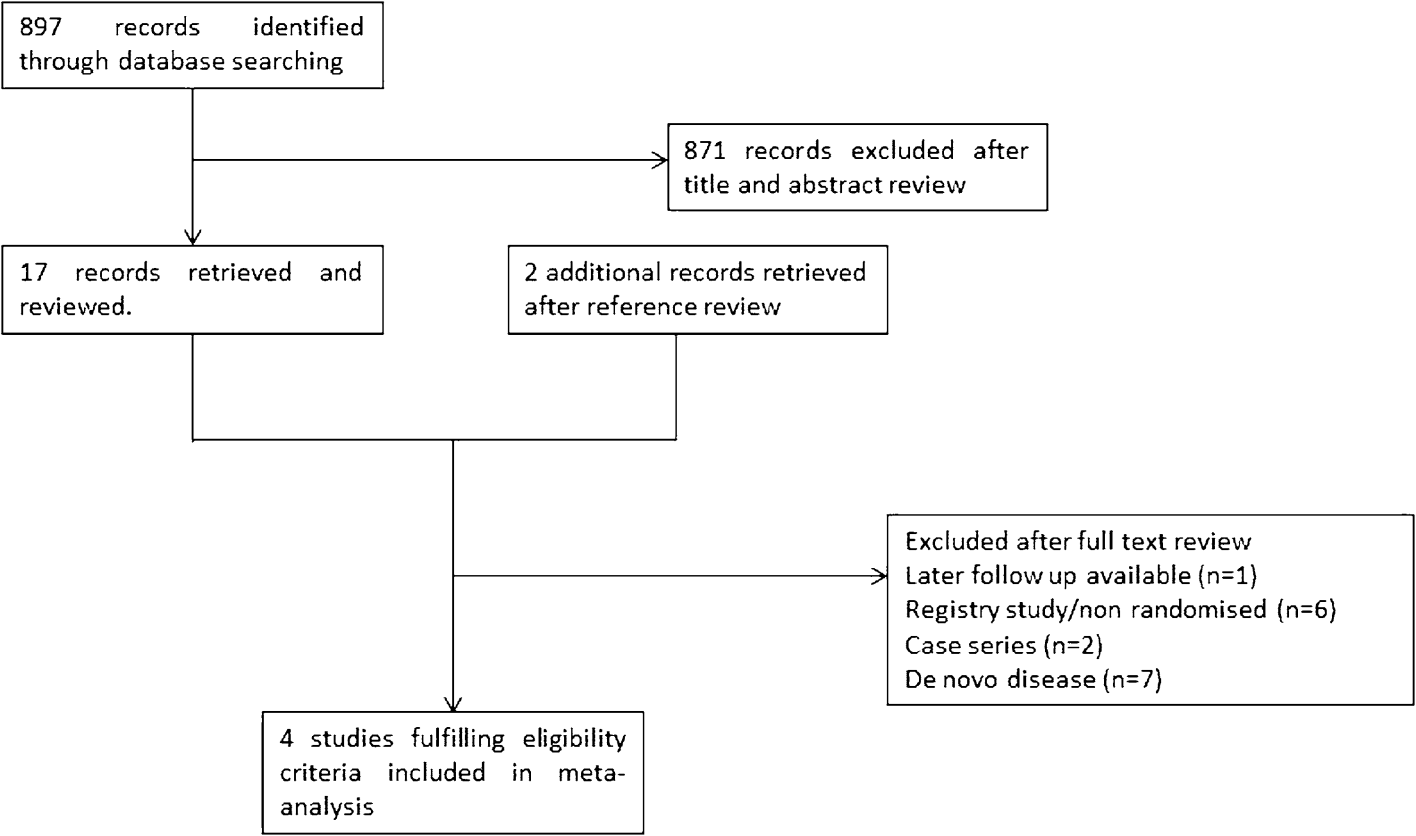
Study flow chart

The initial search identified 897 studies of potential relevance. After assessment of title and abstract, 17 studies were reviewed in full text for eligibility (Fig. 1). Two further studies were identified through review of full text articles. Eight studies were reviewed in full text but not included because they were non-randomised registry studies or case series [7–14]. Further RCTs that focused on different aspects of de novo disease (e.g., bifurcation, MI, small vessels) were also excluded [15–19]. The RCT PACCOCATH I was initially reported at 1-year follow-up [20], but was excluded in favour of the combined PACCOCATH I and II study [21] which had identical protocols and were jointly reported at 2-year follow-up. Thus, four studies were considered eligible for inclusion in the meta-analysis [21–24]. The internal validity of the included studies was appraised by two unblinded reviewers.

### Baseline study characteristics

The studies included in the meta-analysis are summarised in Table 1. Four studies compared DCBs with either conventional balloon angioplasty (POBA) or DES in the treatment of restenosis [21–24]. Three studies used Sequent Please (B. Braun, Melsungen, Germany) [22–24] and one used Paccocath (Bayer Schering Pharma, Berlin, Germany) [21]; both balloons have the same carrier matrix and are coated with 3 μg paclitaxel. Studies were multi-centre in three cases and single centre in one. One study was double-blind, two were single-blind, and one was unblinded. In total, clinical follow-up was available for all 399 patients enroled in the studies of ISR, with angiographic follow-up available for 355 patients (89.0 %). The mean duration of follow-up was 14.5 months.

**Table 1:** Summary of characteristics of randomised control trials included in the meta-analysis

Author/acronym	Years	Setting	Blinding	DCB	Comparator	Lesion characteristic	Total, *n*	Clinical follow-up, *n*		Angiographic follow-up, *n*		Follow-up(months)
								DCB	Control	DCB	Control	
Habara et al. [19]	2011	Single centre	Single	Sequent please	POBA	ISR of SES	50	25	25	23	24	6
PACCOCATH ISR I AND II [18]	2008	Multicentre, Germany	Double	Paccocath	POBA	ISR of BMS or DES	108	54	54	49	48	24
PEPCAD II ISR [20]	2009	Multicentre, Germany	Unblinded	Sequent please	DES^a^	ISR of BMS	131	66	65	57	59	12
PEPCAD-DES [23]	2011	Multicentre, Germany	Single	Sequent please	POBA	ISR of DES	110	72	38	64	31	12

*DCB* drug-coated balloon,* POBA* balloon angioplasty,* DES* drug-eluting stent,* SES* sirolimus-eluting stent,* BMS* bare-metal stent

^a^Taxus Libertè

### Mortality

**Fig. 2:**
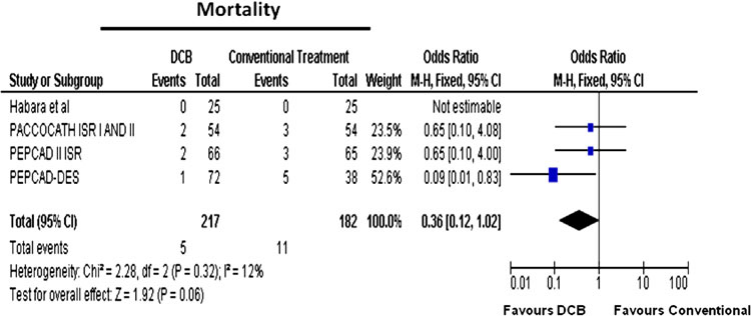
Meta-analysis for mortality in in-stent restenosis for DCB versus control; individual and overall odds ratios of mortality after treatment with DCB or control PCI are reported

The odds ratios (ORs) for mortality in the RCTs of ISR are shown in Fig. 2. There were a total of 16 deaths (Table 2). Mortality rates were numerically lower in DCB-treated patients than in controls with a trend towards statistical significance: the incidence of death was 5/217 (2.3 %) in the DCB group and 11/182 (7.6 %) in the control group [OR (95 % CI) 0.36 (0.12–1.02), *p* = 0.06], as shown in Fig. 2.

**Table 2:** Summary of clinical outcomes in drug-eluting balloon randomised controlled trials

Study	Follow-up (months)	Patients at follow-up		Death (*n*)		MI (*n*)		TLR (*n*)	
		DCB	Control	DCB	Control	DCB	Control	DCB	Control
Habara et al. [19]	6	25	25	0	0	0	0	1	10
PACCOCATH ISR I AND II [18]	24	54	54	2	3	1	5	3	20
PEPCAD II ISR [20]	12	66	65	2	3	0	1	4	10
PEPCAD-DES [23]	12	72	38	1	5	0	1	11	14
Overall		217	182	5	11	1	7	19	54

### Myocardial infarction

**Fig. 3:**
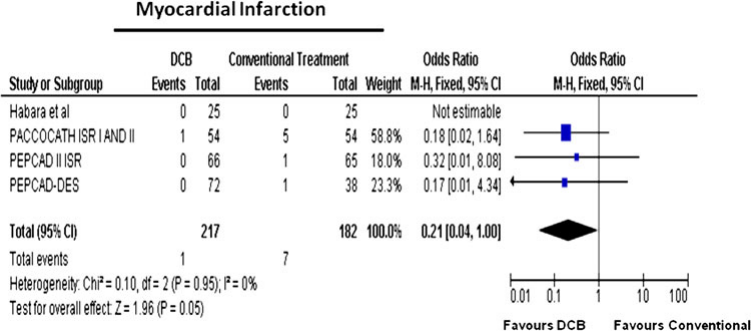
Meta-analysis for myocardial infarction in in-stent restenosis for DCB versus control; individual and overall odds ratios of incident myocardial infarction after treatment with DCB or control PCI are reported

Among the studies of ISR, fewer MIs occurred in the DCB-treated group (Table 2). This finding was of borderline statistical significance: 1/217 (0.5 %) patients in the DCB group and 7/182 (3.8 %) in the control group sustained an MI [OR (95 % CI) 0.21 (0.04–1.00), *p* = 0.05). Figure 3 shows the individual and overall ORs for MI.

### Target lesion revascularisation

**Fig. 4:**
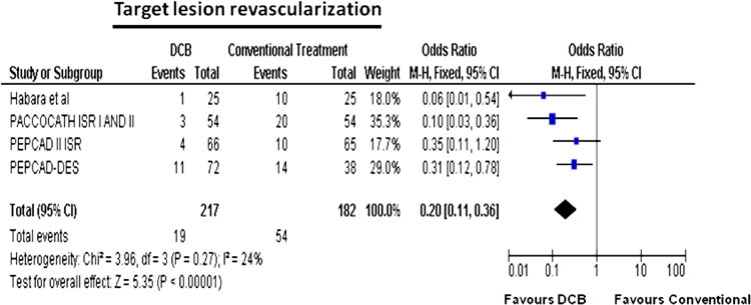
Meta-analysis for target lesion revascularisation in-stent restenosis for DCB versus control: individual and overall odds ratios of target lesion revascularisation after treatment with DCB or control PCI are reported

When compared with controls, DCB use was associated with significantly reduced TLR rates in patients treated for ISR (Table 2): 19/217 (8.8 %) versus 54/182 (29.7 %) [OR (95 % CI)] 0.20 (0.11–0.36), *p* < 0.0001, Fig. 4).

### Binary restenosis and late lumen loss

**Fig. 5:**
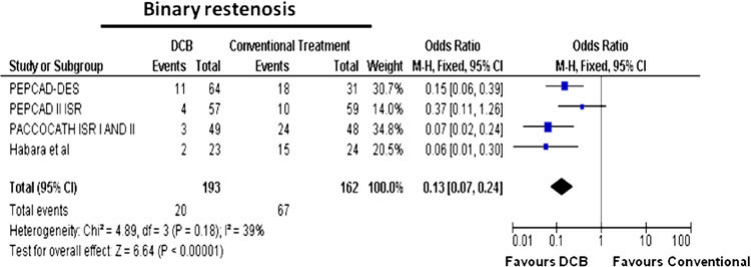
Meta-analysis for binary restenosis in in-stent restenosis for DCB versus control; individual and overall odds ratios of binary restenosis after treatment with DCB or control PCI are reported

**Fig. 6:**
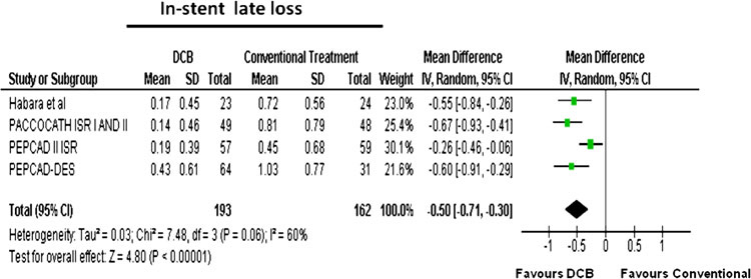
Meta-analysis for in-stent/lesion late luminal loss in in-stent restenosis for DCB versus control; individual and overall means and standard deviations of in-stent/lesion late luminal loss after treatment with DCB or control PCI are reported

As shown in Table 3, the rate of binary restenosis was reduced with the DCB strategy as compared to controls: 20/193 (10.3 %) versus 18/162 (41.3 %). Figure 5 demonstrates that binary restenosis was significantly reduced in DCB-treated patients [OR (95 % CI) 0.13 (0.07–0.24), *p* < 0.00001]. Mean in-stent LLL in the DCB group was 0.23 versus 0.75 mm in the control group (Table 3). Patients treated with DCB had significantly less in-stent LLL than patients receiving control [MD (95 % CI) −0.50 (−0.71; −0.30) mm, *p* < 0.00001, Fig. 6).

**Table 3:** Summary of angiographic outcomes in drug-eluting balloon randomised controlled trials

Study	Angiographic follow-up (*n*)		Binary restenosis		Late luminal loss	
	DCB	Control	DCB	Control	DCB	Control
Habara et al. [19]	23	24	2	15	0.17 ± 0.45	0.72 ± 0.56
PACCOCATH ISR I AND II [18]	49	48	3	24	0.14 ± 0.46	0.81 ± 0.79
PEPCAD II ISR [20]	57	59	4	10	0.19 ± 0.39	0.45 ± 0.68
PEPCAD-DES [23]	64	31	11	18	0.43 ± 0.61	1.03 ± 0.77
Overall	193	162	20	67	0.23	0.75

### Sensitivity analysis

Sensitivity analysis, performed by removing each of the studies one at a time, demonstrated that no single study influenced the overall results. Sensitivity analysis, performed by including each of the studies one at a time according to different length of follow-up, from the lowest to the highest, showed that different follow-up times did not influence the overall results.

### Test for interaction

The interaction test showed no significant difference in TLR results when DCB treatment was compared to control patients treated with either POBA [21, 22, 24] or DES [23] (*χ* ^2^ = 0.54, *df* = 1, *p* = 0.46).

### Publication bias

**Fig. 7:**
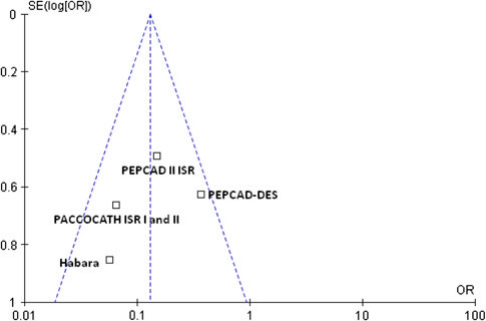
Funnel plot for the mortality outcome. The sample size of each study (measured as standard error of the treatment effect) was plotted against the odds ratio for overall mortality

The funnel plot for mortality did not show asymmetry by visual inspection suggesting no publication bias (Fig. 7); similarly, Egger’s test was not significant, thus excluding the presence of publication bias; the same results were observed for all the chosen outcomes.

### Number needed to treat

**Fig. 8:**
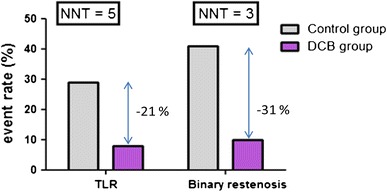
Absolute differences in rates of target lesion revascularisation (TLR) and binary restenosis after treatment with DCB or control PCI for in-stent restenosis

The absolute difference in event rates results in five patients needed to treat to prevent one TLR, and three patients to prevent one binary restenosis (Fig. 8).

## Discussion

The present meta-analysis demonstrates that the use of DCB in the management of ISR is associated with reduction in the incidence of binary restenosis, in-stent LLL, TLR and possibly MI compared with controls (balloon angioplasty or drug eluting stent).

In the United States, DCBs are not currently approved by the regulatory authorities. However, DCBs have a class IIa, level of evidence B indication in the ESC revascularisation guidelines for the management of ISR in bare-metal stent. Recruiting large patient numbers to RCTs for ISR is difficult; as such, individual studies included were relatively small. Meta-analyses in these circumstances are particularly useful. By pooling existing data, this study has provided more robust clinical evidence for a broader group of patients including restenosis of previously implanted DES.

### Management of in-stent restenosis

In-stent restenosis has been reduced but not eliminated by the use of DES. Indeed, given the numbers of more complex interventions with DES, ISR will remain a prevalent clinical presentation and on-going challenge for interventional cardiologists. Current percutaneous treatment options for ISR include treatment with balloon angioplasty (with or without cutting balloons) and the placement of a second (drug-eluting) stent. Vascular brachytherapy has previously been advocated, but is not currently in widespread use. In studies using implantation of a second DES, further re-intervention rates remain high [25]. Thus, there is scope for improved and expanded treatment options in this patient group.

Drug-coated balloons have been studied versus POBA or DES in RCTs of stable patients presenting with restenosis of either a BMS or DES [21–24]. DCB included in the analysis were similar technologies; both balloons have the same carrier matrix and are coated with 3 μg paclitaxel. Overall, less LLL and lower rates of binary restenosis were observed with DCB versus control. The pooled estimates also demonstrated a significant reduction in re-intervention on the target lesion when patients were treated with DCB. Studies included reported clinical follow-up between 6 and 24 months. More recently the PACCOCATH studies showed a durable benefit to 5 years in DCB-treated patients [26].

In the three studies where DCBs were compared with POBA [21, 22, 24], the MD in LLL was remarkably consistent. A single study, PEPCAD II ISR, that compared DCB with a DES (Taxus libertē), showed a trend in favour of DCB but did not achieve statistical significance for a reduction in clinical TLR [23]. Further comparisons with newer generation DES would give additional useful information. PEPCAD II ISR, however, did contribute to the overall impression of superiority for DCB over alternative treatments, with a significant reduction in LLL and numerically fewer binary restenosis and TLR [23]. Furthermore, no interaction with type of control with respect to TLR was demonstrated in the present analysis.

In the management of restenosis, DCBs have the practical advantage of not placing a further layer of metal within a coronary artery and requiring shorter durations of dual anti-platelet therapy. The results of the analysis were consistent, with no evidence of statistical heterogeneity. Therefore, DCBs appear to be an effective choice in the management of ISR.

Perhaps more surprisingly, borderline significant (*p* = 0.05) lower rates of MI were also observed for DCB over controls (1/217 vs. 7/182). The absolute MI rate in this meta-analysis was small, with 8/399 patients reporting an MI. This finding was mainly powered by the PACCOCATH ISR I and II that reported follow-up data at 2 years [21]. A potential explanation for this small but significant absolute difference is that restenosis in itself can manifest as MI. This proportion was estimated at 3.5 % (death or MI) in the TAXUS clinical trials of de novo coronary artery disease, although in unselected practise this has been estimated up to 9.5 % [27, 28]. Based on DES versus BMS RCTs, it has been hypothesised that reducing restenosis can “offset” the impact of late stent thrombosis on the end points of MI and death [27]. Given that no stent thrombosis was recorded, it is possible that DCBs provide the benefit of a reduction in MI by attenuating binary restenosis and LLL, without “trade-off” in this subgroup. More detailed information on the timing of MI in relation to TLR, and a greater number of studied patients and clinical events would be required to confirm this finding and explanation.

### Limitations

In general, the RCTs were well reported and the data were easily abstracted from the published manuscripts. It is recognised as a limitation that the three of the included RCTs were either unblinded [23], or single blind [22, 24]. In addition, the meta-analysis would have been strengthened by the existence of more studies employing DES control.

Many of the included RCTs allowed angiographic rather than purely clinical driven revascularisation of target lesions. RCTs are known in other fields to increase the frequency of TLR clinical end points by the “oculo-stenotic” reflex. It is possible, therefore, that some cases of TLR would not have occurred had protocol angiography not been performed. This limitation in directly applying these research findings to real world clinical practise is mitigated by the important information gained by quantitative angiography in these experimental circumstances.

We opted in this meta-analysis to focus on studies of ISR. This approach has the advantage of comparing treatment in a defined lesion pathology, with studies of similar DCB. It was therefore considered valid to calculate pooled estimates for these studies. However, it could be seen as a limitation that we excluded studies of de novo disease from the current analysis. DCB devices used in some de novo lesion studies have varied significantly in their design to current devices (e.g., no carrier molecule, “DCB-facilitated” BMS), which in some cases have not been made available for use due to lack of efficacy [16, 29]. Furthermore, for those remaining DCB tested in RCTs, both the patient sub-groups (e.g. AMI, stable angina), and comparator stents (e.g. DES, EPC capture stents) were incongruent, rendering a comparison limited at best [17]. Thus, it was concluded that a fair comparison is not currently possible. DCBs remain in a developmental stage in de novo disease, and further RCTs are awaited.

Finally, a limitation of this meta-analysis, common to all the meta-analyses based on study-level data, is the lack of individual patient data that would have further improved the results. However, an in-depth robust statistical analysis in the present study revealed no heterogeneity or publication bias. Further, given our study does not contain patient level data, it is not possible to determine if there were differences in the degree of angina pectoris and dyspnoea before and following DCB treatment.

## Conclusion

Drug-coated balloons appear effective versus controls (balloon angioplasty or DES) in improving angiographic outcomes, reducing TLR and possibly MI when used in the management of restenosis of previously implanted stents.

## Abbreviations

BMS: Bare-metal stent
CI: Confidence interval
DCB: Drug-coated balloon
DES: Drug-eluting stent
EPC: Endothelial progenitor cell
ISR: In-stent restenosis
LLL: Late luminal loss
MD: Mean difference
MI: Myocardial infarction
OR: Odds ratio
POBA: Balloon angioplasty
PACCOCATH ISR: Treatment of coronary in-stent restenosis with a catheter
PCI: Percutaneous coronary intervention
PEPCAD II: Paclitaxel-coated balloon catheter versus paclitaxel-coated stent for the treatment of coronary in-stent restenosis
PEPCAD DES: Prospective randomized trial of a paclitaxel-coated balloon vs. uncoated balloon angioplasty in patients with drug-eluting stent restenosis
PEPPER: Paclitaxel releasing balloon in patients presenting with in-stent restenosis
RCT: Randomised controlled trial
TLR: Target lesion revascularisation
